# Transverse-electric plasmonic modes of cylindrical graphene-based waveguide at near-infrared and visible frequencies

**DOI:** 10.1038/srep26915

**Published:** 2016-05-26

**Authors:** Dmitry A. Kuzmin, Igor V. Bychkov, Vladimir G. Shavrov, Leonid N. Kotov

**Affiliations:** 1 Chelyabinsk State University, Department of Radio-Physics and Electronics Chelyabinsk, 454001, Russian Federation; 2 South Ural State University (National Research University), Chelyabinsk, 454080, Russian Federation; 3 Kotelnikov Institute of Radio-engeneering and Electronics of RAS, Laboratory of magnetic phenomena in microelectronics, Moscow, 125009, Russian Federation; 4 Syktyvkar State University named after Pitirim Sorokin, Syktyvkar, 167001, Russian Federation

## Abstract

Transverse-electric (TE) surface plasmons (SPs) are very unusual for plasmonics phenomenon. Graphene proposes a unique possibility to observe these plasmons. Due to transverse motion of carriers, TE SPs speed is usually close to bulk light one. In this work we discuss conditions of TE SPs propagation in cylindrical graphene-based waveguides. We found that the negativity of graphene conductivity’s imaginary part is not a sufficient condition. The structure supports TE SPs when the core radius of waveguide is larger than the critical value *R*_*cr*_. Critical radius depends on the light frequency and the difference of permittivities inside and outside the waveguide. Minimum value of *R*_*cr*_ is comparable with the wavelength of volume wave and corresponds to interband carriers transition in graphene. We predict that use of multilayer graphene will lead to decrease of critical radius. TE SPs speed may differ more significantly from bulk light one in case of epsilon-near-zero core and shell of the waveguide. Results may open the door for practical applications of TE SPs in optics, including telecommunications.

Graphene, two-dimensional honey-comb-like carbon lattice, is a very promising material for many optics, plasmonics and photonics applications^1^,^2^,^3^. In contrary to metal based plasmonics, graphene may support TE-polarized plasmon-polaritons as well, as TM-polarized ones^4^,^5^,^6^. TE plasmon-polaritons in suspended graphene are close to the light line and weakly localized. Such plasmon-polaritons exist when imaginary part of graphene conductivity is negative *Im*[*σ*_*g*_] < 0, what corresponds to the frequency range defined by the following: 1.667 < *ħω*/*μ*_*ch*_ < 2, where *ħ* is Plank constant, *ω* = 2*πf* is the angular frequency (time dependence exp-*iωt*]), *μ*_*ch*_ is the chemical potential (or Fermi level) of graphene. Chemical potential is defined by charge-carrier concentration *n*: *μ*_*ch*_ ≈ *ħv*_*F*_(*πn*)^1/2^, where *v*_*F*_ ≈ 10^6^ m/s is the Fermi velocity. Charge-carrier concentration may be tuned by chemical doping or electrostatic bias^7^,^8^,^9^. Nowadays, gate voltage allows reaching Fermi level values up to about 1 eV (or, equivalently, charge-carrier concentration ~7 · 10^13^ cm^−2^). While TM plasmons exist at *ħω*/*μ*_*ch*_ < 1.667, which correspond to frequencies up to mid-infrared range, TE ones may be excited at higher frequencies up to visible light through telecommunication frequencies. This fact makes TE plasmons very promising for practical applications in communication technologies. Due to the small spectral range, TE-polarized plasmons have been directly observed in experiment only recently^10^. In bilayer graphene it is expected stronger field confinement of this mode^11^. In two-layer configuration at certain spacer properties both symmetric and anti-symmetric modes may exist^12^.

Indeed, only graphene ribbons may be used in planar geometry. Edges of the ribbons lead usually to increase of loss^13^. To avoid undesirable losses one may use cylindrical structures in real plasmonic devices^14^. TM plasmonic modes of cylindrical graphene-based waveguides were investigated in^15^. Graphene-based cylindrical waveguides have been analyzed in different configurations^16^, the single-mode realization of such waveguide was proposed^17^. In case of magnetically-biased graphene coating authors have paid attention to hybridization of TE and TM plasmonic modes. Nevertheless, conditions of supporting all-TE plasmonic modes by cylindrical graphene-based waveguide have never been stated yet. By the other hand, TE modes are more susceptible to radiation from the edges due to their proximity to light cone. Thus, cylindrical structures may be more suitable for practical applications of TE plasmons.

## Results

**Figure 1:**
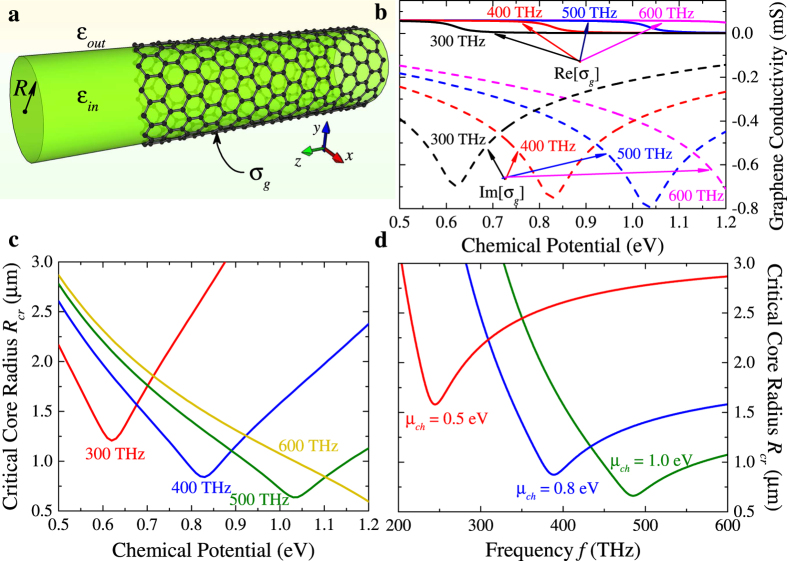
Geometry of the problem (**a**), conductivity of graphene versus chemical potential at different frequencies (**b**), critical core radius versus graphene chemical potential (**c**) and frequency (**d**).

Geometry of the problem is shown in Fig. 1a. Dielectric cylinder (core of the waveguide) with dielectric permittivity 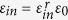 (we use SI units, *ε*_0_ is electric constant) and radius *R*, is coated by graphene layer. Such cylinder is embedded in dielectric medium with dielectric permittivity 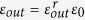. Both mediums are non-magnetic (*μ*_*in*_ = *μ*_*out*_ = *μ*_0_). the cylinder axis coincides with *z*-axis. For modeling graphene optical properties we used the standard procedure (see Methods for details). Dependencies of real and imaginary parts of graphene conductivity from its chemical potential for some frequencies are shown in Fig. 1b.

In cylindrical geometry, only fundamental modes (i.e. *ϕ*-independent) may be classified into TE- and TM- polarized. Higher modes are hybrid TE-TM ones. Depending on relation between longitudinal field components *E*_*z*_ and *H*_*z*_, one may divide modes into quasi-TE and quasi-TM polarized. Due to hybrid nature of such modes, their dispersion equations are equals.

Using appropriate boundary conditions, one may obtain dispersion equation of fundamental TE mode (see Methods for details):


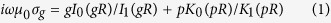


In (1) *I*_*n*_(*gr*) and *K*_*n*_(*pr*) are modified Bessel functions of *n*-th order, 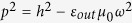, 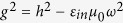.

For further analysis let us limit ourselves by the case of real values of *p* and *g*. Both terms in right-hand side of equation (1) are positive, so, we may conclude that condition Im [*σ*_*g*_] < 0 is necessary. But this condition is not sufficient. Let us suppose that 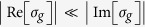. This condition is good satisfied near the interband transition (i.e. when 1.667 < *ħω*/*μ*_*ch*_ < 2). Let us put *iσ*_*g*_ ≈ |Im[*σ*_*g*_]|. Cut-off limit (i.e. *p* → 0) leads to critical coupling between radius of the core, frequency and dielectric permittivity:


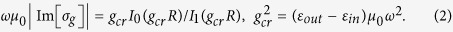


Let us consider two limit cases. The first one, when *g*_*cr*_*R* ≪ 1, corresponds to small core radius, small difference of permittivities of the inner and the outer mediums, or low frequency. This limit leads to the following condition:


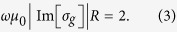


In case of small radius and low frequencies equation (3) and condition *g*_*cr*_*R* ≪ 1 can not be satisfied simultaneously. There is only one way: to reduce the difference (*ε*_*out*_ − *ε*_*in*_). Using the limitation condition 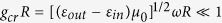 with equation (3), one may obtain the following


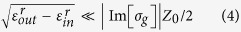


where we use relative permittivities 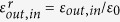, and *Z*_0_ = (*μ*_0_/*ε*_0_)^1/2^ is the impedance of free space. Taking into account that in frequency range 100–600 THz (near infrared to visible light, wavelegth range from 500 nm to 3 *μ*m) |Im[*σ*_*g*_]| may reach (at *ħω*/*μ*_*ch*_ ≈ 2) up to 10^−4^–10^−3 ^S, one can estimate 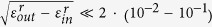. So, equation (3) may be used for estimation of the minimal radius only in case of two almost identical mediums. Estimations of critical radius from equation (3) give *R* ∼ 1 *μ*m. We should note that in other cases, the critical radius is greater.

In the opposite case, when *g*_*cr*_*R* ∼ 1, one may obtain:


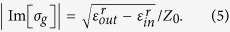


Estimating from (5) *g*_*cr*_*R* = [(*ε*_*out*_ − *ε*_*in*_)*μ*_0_]^1/2^*ωR* ≈ 2 · (10^−2^–10^−1^)*ωR*/*c*, one may conclude, that the case under consideration will be realized when *ωR* ≫ 1.5 · (10^9^–10^10^) m/s. For the frequencies 100–600 THz, we will have *R* ≫ 1 *μ*m. This situation is poorly suitable for practical applications and will not be considered further.

In the intermediate case, when *g*_*cr*_*R* ≈ 1, we will have 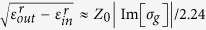. Numerical estimations shows that 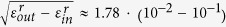. We should note that such case may be realized only at fixed permittivity difference and core radius, given by 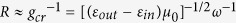. This is very difficult for practical realization.

Let us consider more detailed the case of equal core and outer mediums, which correspond to the lowest core radius. Dependencies of the critical radius, calculated from equation (3), versus the frequency of propagating waves and graphene chemical potential are shown in Fig. 1c,d.

One can see that the minimal value of critical radius corresponds to intrerband transitions of carriers in graphene *ħω*/*μ*_*ch*_ ≈ 2. This value is comparable with the wavelength of the volume wave. Increasing of the frequency at fixed chemical potential leads to decrease of minimal value of critical radius. At fixed core radius, range of chemical potentials, when the structure supports TE plasmons, increases with increasing of frequency. *Vice versa*, increase of chemical potential leads to increase of the frequency range. We should to note, that at certain values of core radius the waveguide may support TE plasmons in wide frequency range. For example, at *R* = 1.25 *μ*m for chemical potential 1.0 eV the frequency window is from about 425 THz to more than 600 THz, at *R* = 1.75 *μ*m for chemical potential 0.8 eV–from about 330 THz, at *R* = 3 *μ*m for chemical potential 0.5 eV–from about 200 THz. Thus, proposed structure may work as a high pass filter with controllable characteristics.

**Figure 2:**
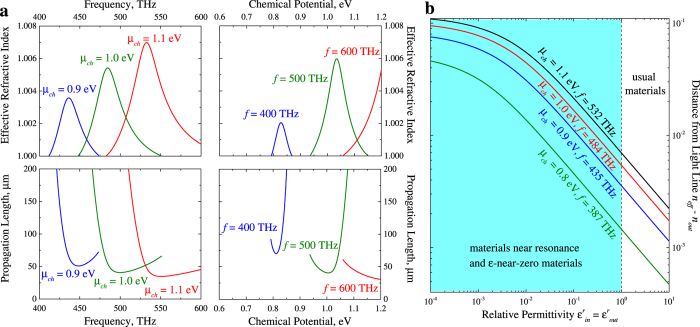
Characteristics of guiding TE plasmon-polaritons (**a**) and distance of TE plasmon mode from light line (**b**). Core radius *R* = 1 *μ*m. Permittivities of the core and the outer mediums are equals. Filled region in (**b**) correspond to the values 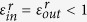, what may be observed in some materials at near the resonance conditions or in *ε*-near-zero materials and metamaterials, logarithmic scale is used for both axes.

Main characteristics of propagating plasmon-polaritons are the effective refractive index *n*_*eff*_ = Re[*h*]/*k*_0_, where *k*_0_ = *ω*/*c*, and propagation length *L*_*SPP*_ = 1/(2Im[*h*]). Let us investigate these characteristics for guiding TE mode. For simplicity, we will put *ε*_*out*_ = *ε*_*in*_ = *ε*_0_. Core radius will be *R* = 1 *μ*m. At such core radius, the structure may support TE plasmon-polaritons in frequency range from approximately 370 THz and chemical potential values from approximately 0.8 eV. Figure 2a shows dependencies of *n*_*eff*_ and *L*_*SPP*_ from frequency and chemical potential calculated from exact dispersion equation given by (1).

One can see, that the maximal effective refractive index corresponds to condition *ħω*/*μ*_*ch*_ ≈ 2. In the case under consideration localization of plasmonic mode is characterized by parameter 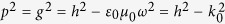. So, exceeding the unity by the effective refractive index describes the mode localization. TE mode of cylindrical graphene waveguide is weakly localized, similarly to the case of suspended single layer graphene^4^,^9^. The propagation length at *ħω*/*μ*_*ch*_ ≈ 2 is close to the minimal value due to higher localization. At fixed frequency (chemical potential) propagation length increase rapidly when chemical potential (frequency) decrease (increase), while localization is almost the same. This is due to decrease of real part of graphene conductivity at *ħω*/*μ*_*ch*_ < 2, and hence to decrease of dissipation.

Similarly to the single layer graphene and the double-layer configuration^12^,^18^, TE mode in proposed structure is very sensitive to difference between dielectric permittivities of the core and the outer medium. Calculations show that at more favorable for TE mode propagation condition of *ħω*/*μ*_*ch*_ ≈ 2, the structure may support this mode only while 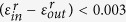 for *μ*_*ch*_ = 0.8 eV (*f* ≈ 387 THz), 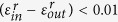 for *μ*_*ch*_ = 0.9 eV (*f* ≈ 435 THz), 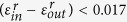 for *μ*_*ch*_ = 1.0 eV (*f* ≈ 484 THz), and 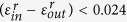 for *μ*_*ch*_ = 1.1 eV (*f* ≈ 532 THz). Our estimations give smaller critical values of difference between dielectric permittivities than those for double-layer configuration (for the spacer thickness 100 nm critical 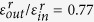
12, while we have 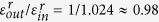). This is caused by the fact, that fundamental TE mode of cylindrical waveguide is better comparable with symmetric mode in planar geometry, which has not been considered in details.

The main difficulty for experimental detection and practical applications of TE plasmons in graphene is their proximity to the light line. The distance of TE modes from the light line may be described by the difference 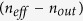, where 
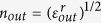
 is the refractive index of the outer medium. As we have mentioned above, the most favorable for TE mode propagation conditions are *ħω*/*μ*_*ch*_ = 2 and 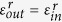. Let us investigate how the value of permittivity will affect characteristics of TE modes. Dependence of the plasmon distance from the light line 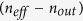 from the permittivity for different values of chemical potential and frequency, corresponding to the condition of interband transitions, is shown in Fig. 2b. Core radius is equal to 1 *μ*m. Filled region correspond to the values 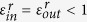. Such situation may be observed in some materials at near the resonance conditions or in *ε*-near-zero materials and metamaterials^19^,^20^. One can see, that at values of permittivity 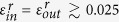 plasmon distance from the light line 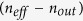 decrease almost exponentially with increasing of permittivity (logarithmic scale is used for both axes in Fig. 2b). At 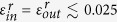 the difference 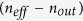 increase slowly with decrease of permittivity. Comparing the values of 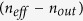 for usual materials (*ε*^*r*^ > 1) with the ones for materials at near resonance conditions and *ε*-near-zero materials, one may conclude that in the last case the difference 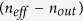 may be in order of magnitude greater. So, for experimental observation of TE modes may be more favorable to use *ε*-near-zero metamaterials.

## Discussion

Let us discuss now the possible ways to reduce the critical radius. From equation (3) one can see that there is only one way–to increase the absolute value of imaginary part of the surface conductivity. This purpose may be achieved by use of few-layer graphene. Detailed investigation has shown^11^ that in bilayer graphene TE plasmons may departs more from light line comparing with the single graphene layer. An effective optical conductivity of randomly oriented few-layer graphene is proportional to the number of layers *N*^21^,^22^,^23^,^24^. Thus, the absolute value of imaginary part of conductivity will increase, and critical radius value will decrease approximately *N* times. For example, if one will use the 5 layer graphene, all results we have obtained for the core radius of 1 *μ*m, may be observed at core radius of about 200 nm. Experimentally, TE-plasmons have been observed at telecommunication frequencies^10^ (wavelength in vacuum is 1.55 *μ*m). With taking into account the possibility of reducing the critical core radius, proposed structure may have wide perspectives in communication technologies.

High-order modes of the waveguide under investigation are described by the same dispersion equation, which was obtained by Gao *et al.*^15^ due to hybrid TE-TM fields structure. At interband transitions condition TE component will dominate in such modes. Calculations show that such modes may be supported by the structure at much larger core radius. For example, for *μ*_*ch*_ = 1.0 eV (*f* ≈ 484 THz) and equal core and outer mediums the structure may support the mode with azimuthal distribution ~exp[*iϕ*] only at core radius of about some centimeters. This is not appropriate for practical applications. By the other hand, this fact shows that the proposed structure may work in single-mode regime in wide frequency range.

In conclusions, we have investigated TE plasmonic modes supported by cylindrical graphene based structure. Such modes may propagate in the structure only at core radius larger than at least 500 nm at frequencies of near-infrared-to-visible light. The most favorable condition for TE plasmons propagation is the equality of the core and the outer mediums. TE plasmons dispersion departs from the light line more significantly for low values of permittivities 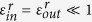. Use of few-layer graphene should lead to decrease of critical radius or operating frequency. Results have a potential interest for practical design of optical devices based on TE plasmonic modes of cylindrical graphene waveguides in wide frequency range, including telecommunication frequencies.

## Methods

We used cylindrical coordinates (*r*, *ϕ*, *z*). For obtain dispersion equation, one should to solve Maxwell’s equations inside each medium with taking into account the boundary conditions: 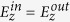, 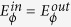, 
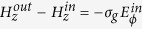
, and 
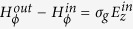
. Considering the waves propagating along cylinder axis, one may put electric and magnetic fields **E**, **H** ~ exp[−*iωt* + *ihz*].

Graphene layer was modeled as infinitesimally thin surface with the surface conductivity *σ*_*g*_, calculated from Kubo formula^25^,^26^,^27^. At finite temperature it may be divided into intra- and interband contributions *σ*_*g*_ = *σ*_*intra*_ + *σ*_*inter*_:


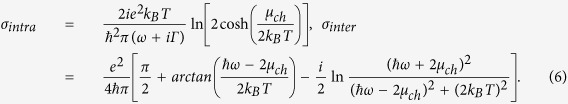


In (6) *T* is the temperature, Γ is the charge carriers scattering rate. For numerical calculations we will use *T* = 300 K, and Γ = 0.1 meV.

For fundamental TE mode, field components may be obtained from common field distribution:


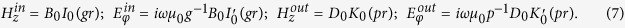


Here, *I*_0_(*gr*) and *K*_0_(*pr*) are modified Bessel functions, prime denotes the derivative with respect to the argument, 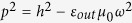, 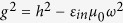.

Using these field expressions with boundary conditions, one may obtain a system of equations with respect to constants *B*_0_ and *D*_0_:


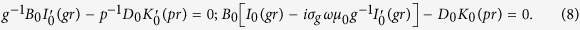


By equating the determinant of the matrix constructed from coefficients at unknown constants, one may obtain dispersion equation (1).

## Additional Information

**How to cite this article**: Kuzmin, D. A. *et al.* Transverse-electric plasmonic modes of cylindrical graphene-based waveguide at near-infrared and visible frequencies. *Sci. Rep.* **6**, 26915; doi: 10.1038/srep26915 (2016).

## References

[b1] Vakil A. & Engheta N. Transformation optics using graphene. Science 332, 1291–1294 (2011). 21659598 10.1126/science.1202691

[b2] Grigorenko A. N., Polini M. & Novoselov K. S. Graphene plasmonics. Nature Photonics 6, 749–758 (2012).

[b3] Bao Q. & Loh K. P. Graphene photonics, plasmonics, and broadband optoelectronic devices. ACS Nano 6, 3677–3694 (2012). 22512399 10.1021/nn300989g

[b4] Mikhailov S. A. & Ziegler K. New electromagnetic mode in graphene. Physical Review Letters 99, 016803 (2007). 17678180 10.1103/PhysRevLett.99.016803

[b5] Bludov Y. V., Ferreira A., Peres N. M. R. & Vasilevskiy M. I. A primer on surface plasmon-polaritons in graphene. International Journal of Modern Physics B 27, 1341001 (2013).

[b6] Xiao S., Zhu X., Li B. H. & Mortensen N. A. Graphene-plasmon polaritons: From fundamental properties to potential applications. Frontiers of Physics 11, 117801 (2016).

[b7] Gusynin V. P. & Sharapov S. G. Transport of dirac quasiparticles in graphene: Hall and optical conductivities. Physical Review B 73, 245411 (2006). 10.1103/PhysRevLett.96.256802 16907333

[b8] Gusynin V. P., Sharapov S. G. & Carbotte J. P. Magneto-optical conductivity in graphene. Journal of Physics Condensed Matter 19, 026222 (2007).

[b9] Hanson G. W. Dyadic Green’s functions and guided surface waves for a surface conductivity model of graphene. Journal of Applied Physics 103, 064302 (2008).

[b10] Menabde S. G., Mason D. R., Kornev E. E., Lee C. & Park N. Direct optical probing of transverse electric mode in graphene. Scientific Reports 6, 21523 (2016). 26898892 10.1038/srep21523 PMC4761906

[b11] Jablan M., Buljan H. & Soljačić M. Transverse electric plasmons in bilayer graphene. Optics Express 19, 11236–11241 (2011). 21716353 10.1364/OE.19.011236

[b12] Buslaev P. I., Iorsh I. V., Shadrivov I. V., Belov P. A. & Kivshar Y. S. Plasmons in waveguide structures formed by two graphene layers. JETP Letters 97, 535–539 (2013).

[b13] Yan H. *et al.* Damping pathways of mid-infrared plasmons in graphene nanostructures. Nature Photonics 7, 394–399 (2013).

[b14] Lamata I. S., Alonso-Gonzalez P., Hillenbrand R. & Nikitin A. Y. Plasmons in cylindrical 2d materials as a platform for nanophotonic circuits. ACS Photonics 2, 280–286 (2015).

[b15] Gao Y. *et al.* Analytical model for plasmon modes in graphene-coated nanowire. Optics Express 22, 24322–24331 (2014). 25322007 10.1364/OE.22.024322

[b16] Correas-Serrano D., Gomez-Diaz J. S., Alu A. & Alvarez-Melcon A. Electrically and magnetically biased graphene-based cylindrical waveguides: Analysis and applications as reconfigurable antennas. IEEE Transactions on Terahertz Science and Technology 5, 951–960 (2015).

[b17] Gao Y., Ren G., Zhu B., Wang J. & Jian S. Single-mode graphene-coated nanowire plasmonic waveguide. Optics Letters 39, 5909–5912 (2014). 25361117 10.1364/OL.39.005909

[b18] He X. Y., Tao J. & Meng B. Analysis of graphene TE surface plasmons in the terahertz regime. Nanotechnology 24, 345203 (2013). 23912303 10.1088/0957-4484/24/34/345203

[b19] Maas R., Parsons J., Engheta N. & Polman A. Experimental realization of an epsilon-near-zero metamaterial at visible wavelengths. Nature Photonics 7, 907–912 (2013).

[b20] Moitra P. *et al.* Realization of an all-dielectric zero-index optical metamaterial. Nature Photonics 7, 791–795 (2013).

[b21] Hass J. *et al.* Why multilayer graphene on 4h-SiC(0001) behaves like a single sheet of graphene. Physical Review Letters 100, 125504 (2008). 18517883 10.1103/PhysRevLett.100.125504

[b22] Dawlaty J. M. *et al.* Measurement of the optical absorption spectra of epitaxial graphene from terahertz to visible. Applied Physics Letters 93, 131905 (2008).

[b23] Yan H. *et al.* Infrared spectroscopy of wafer-scale graphene. ACS Nano 5, 9854–9860 (2011). 22077967 10.1021/nn203506n

[b24] Baek I. H. *et al.* Terahertz transmission and sheet conductivity of randomly stacked multi-layer graphene. Applied Physics Letters 102, 191109 (2013).

[b25] Falkovsky L. A. & Varlamov A. A. Space-time dispersion of graphene conductivity. The European Physical Journal B 56, 281–284 (2007).

[b26] Falkovsky L. A. & Pershoguba S. S. Optical far-infrared properties of a graphene monolayer and multilayer. Physical Review B 76, 153410 (2007).

[b27] Falkovsky L. A. Optical properties of graphene and IV–VI semiconductors. Physics-Uspekhi 51, 887–897 (2008).

